# Insights into the evolution of enzyme substrate promiscuity after the discovery of (βα)_8_ isomerase evolutionary intermediates from a diverse metagenome

**DOI:** 10.1186/s12862-015-0378-1

**Published:** 2015-06-10

**Authors:** Lianet Noda-García, Ana L. Juárez-Vázquez, María C. Ávila-Arcos, Ernesto A. Verduzco-Castro, Gabriela Montero-Morán, Paul Gaytán, Mauricio Carrillo-Tripp, Francisco Barona-Gómez

**Affiliations:** Evolution of Metabolic Diversity, Unidad de Genómica Avanzada (Langebio), Cinvestav-IPN, Km 9.6 Libramiento Norte, Carretera Irapuato - León, CP 36821 Irapuato, México; Biomolecular Diversity Laboratories, Unidad de Genómica Avanzada (Langebio), Cinvestav-IPN, Km 9.6 Libramiento Norte, Carretera Irapuato - León, CP 36821 Irapuato, México; Instituto de Biotecnología, Universidad Nacional Autónoma de México (UNAM), Av. Universidad 2001, CP 62250 Cuernavaca, México; Current Addresses: Department of Biological Chemistry, Weizmann Institute of Science, Rehovot, Israel; Current Addresses: División de Biología Molecular, Instituto Potosino de Investigación Científica y Tecnológica, San Luis Potosí, México; Current Addresses: Department of Genetics, Stanford University, Stanford, CA USA

## Abstract

**Background:**

Current sequence-based approaches to identify enzyme functional shifts, such as enzyme promiscuity, have proven to be highly dependent on *a priori* functional knowledge, hampering our ability to reconstruct evolutionary history behind these mechanisms. Hidden Markov Model (HMM) profiles, broadly used to classify enzyme families, can be useful to distinguish between closely related enzyme families with different specificities. The (βα)_8_-isomerase HisA/PriA enzyme family, involved in L-histidine (HisA, mono-substrate) biosynthesis in most bacteria and plants, but also in L-tryptophan (HisA/TrpF or PriA, dual-substrate) biosynthesis in most *Actinobacteria*, has been used as model system to explore evolutionary hypotheses and therefore has a considerable amount of evolutionary, functional and structural knowledge available. We searched for functional evolutionary intermediates between the HisA and PriA enzyme families in order to understand the functional divergence between these families.

**Results:**

We constructed a HMM profile that correctly classifies sequences of unknown function into the HisA and PriA enzyme sub-families. Using this HMM profile, we mined a large metagenome to identify plausible evolutionary intermediate sequences between HisA and PriA. These sequences were used to perform phylogenetic reconstructions and to identify functionally conserved amino acids. Biochemical characterization of one selected enzyme (CAM1) with a mutation within the functionally essential N-terminus phosphate-binding site, namely, an alanine instead of a glycine in HisA or a serine in PriA, showed that this evolutionary intermediate has dual-substrate specificity. Moreover, site-directed mutagenesis of this alanine residue, either backwards into a glycine or forward into a serine, revealed the robustness of this enzyme. None of these mutations, presumably upon functionally essential amino acids, significantly abolished its enzyme activities. A truncated version of this enzyme (CAM2) predicted to adopt a (βα)_6_-fold, and thus entirely lacking a C-terminus phosphate-binding site, was identified and shown to have HisA activity.

**Conclusion:**

As expected, reconstruction of the evolution of PriA from HisA with HMM profiles suggest that functional shifts involve mutations in evolutionarily intermediate enzymes of otherwise functionally essential residues or motifs. These results are in agreement with a link between promiscuous enzymes and intragenic epistasis. HMM provides a convenient approach for gaining insights into these evolutionary processes.

**Electronic supplementary material:**

The online version of this article (doi:10.1186/s12862-015-0378-1) contains supplementary material, which is available to authorized users.

## Background

Numerous reports have shown that enzyme promiscuity, defined as the capacity of an enzyme to perform activities other than the function for which they have evolved–using the same active site– is an extremely common event [1, 2]. The biological implications of these ‘secondary’ activities have been broadly discussed at different levels. The redundancy of enzymatic functions has been hypothesized to lead to a ‘plastic’ metabolic network, important for organismal evolution [3]. At the protein level, these secondary activities have been hypothesized to serve as raw material for the evolution of new activities [4]. Thus, promiscuity could represent an evolutionary advantage that could be selected for as part of a mechanism to acquire novel enzyme functions [2, 5, 6]. Indeed, not only positive selection but also neutral evolution, which leads to accumulation of non-conserved mutations usually away from catalytic active sites, have been proposed to lead to promiscuous enzymes [7].

Methods for functional classification of protein sequence data based on molecular evolution theory assume that members of protein families will diverge from the consensus sequence as functional shifts take place. However, the sequence differences associated with such functional shifts, mainly at early evolutionary stages when enzyme promiscuity and sign epistasis occur [8–11], are difficult to detect. Moreover, the contribution of point mutations may vary broadly both in terms of the derived trade-off between promiscuous and primary activities [12, 13] and their closeness to the active site of enzymes [14, 15]. Therefore, our inability to predict enzyme promiscuity, whose evolutionary nature remains to be fully understood, hampers current enzyme classification systems.

The abovementioned conundrum relates to the issue of ‘hidden’ or adjacent information in biology, a recurring theme in many fields with heterogeneous large datasets. Such heterogeneity has been probabilistically tackled using Hidden Markov Models (HMM) [16, 17]. HMM profiles, a broadly used method for sequence classification and protein functional annotation (e.g. Pfam), can be defined as a motif definition with some probabilities involved. Indeed, a recent study partly based on the usage of HMM profiles shows how these tools can yield important information on the origin of enzymatic functions [18].

**Fig. 1:**
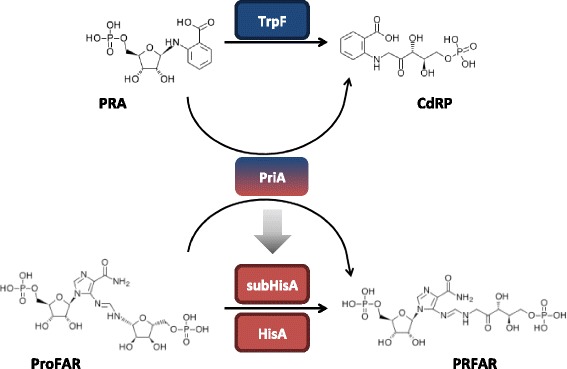
Enzymatic activities of the HisA / PriA enzyme superfamily. HisA is a ubiquitous enzyme that catalyzes the conversion of *N’*-[(5*′*-phosphoribosyl) formimino]-5-aminoimidazole-4-carboxamide ribonucleotide (ProFAR) into N’-[(5′-phosphoribulosyl) formimino]-5-aminoimidazole-4-carboxamide ribonucleotide (PRFAR). PriA, exclusively found in the *Actinobacteridae* class of the high G + C Gram positive bacteria, evolved from HisA to catalyze, in addition to the HisA activity, the conversion of phosphoribosyl anthranilate (PRA) into 1-[(2-carboxyphenyl)amino]-1-deoxyribulose 5-phosphate (CdRP). This latter activity is the same as that catalyzed by the ubiquitous TrpF enzyme. subHisA has recently evolved from PriA (indicated with a gray arrow), and it is found exclusively in certain, but not all, *Corynebacterium* species. As with HisA, subHisA is mono-functional enzyme

Here we hypothesized that HMM profiles provide an efficient approach to identify subtle functional shifts involving enzyme promiscuity. To test this we focus on the (βα)_8_-isomerase HisA or *N*’-[(5′-phosphoribosyl)formimino]-5-aminoimidazole-4-carboxamide ribonucleotide (ProFAR) isomerase (*hisA* gene, EC 5.3.1.16) enzyme family (Fig. 1). HisA is involved in L-Histidine biosynthesis, and due to its internal symmetry, it has been largely used to test hypotheses related to the evolution of the (βα)_8_-fold from smaller [(βα)_2_, (βα)_3_, (βα)_4_, (βα)_6_] subunits [19–25]. Moreover, the HisA homologs from the class *Actinobacteria* have been renamed as PriA, from Phosphoribosyl Isomerase A [26], due to their broader specificity. Indeed, in addition to their ancestral HisA activity, PriA enzymes also have a proficient *N’*-(5′-phosphoribosyl) anthranilate (PRA) isomerase or TrpF activity (*trpF* gene, EC 5.3.1.24) (Fig. 1). Thus, where a *trpF* gene is lacking, as in the *Actinobacteria* or Gram positive eubacteria with high (G + C) content, PriA participates in the biosynthesis of both L-histidine and L-tryptophan.

In addition to PriA, we have recently reported a closely related group of homologs specific to a certain sub-clade of the genus *Corynebacterium*, which was renamed as subHisA [27]. The subHisA enzyme sub-family evolved from PriA in a process resembling sub-functionalization, and it lacks any TrpF activity, rendering a mono-substrate ProFAR specific HisA-like enzyme. The HisA, PriA and subHisA enzyme sub-families share 35 % sequence identity between them, and several of their catalytic amino acid residues have been identified (see reference 25 for a review). Notably, at least one residue that is conserved at the sub-family level, Gly81, Ser81 and Thr81, respectively, was identified early on and further characterized [28]. More recently, additional PriA [30] and subHisA [27] specific residues, including Arg143 and Asn142, respectively, have been discovered as sub-family specific residues. These residues are part of the N-terminal phosphate-binding site (PBS) motif, where substrate specificity takes place, in equivalent positions within the (βα)_8_-fold adopted by these enzymes.

Despite these well-established sequence, structural, and functional differences, semi-automatic annotation methods based in multiple sequence alignments (MSA) and structural similarities, such as those used by the SCOP and CATH databases, continue to group some HisA, PriA and subHisA sequences within one single enzyme family, and under the same Enzyme Classification (EC) number. For instance, at time of submission of this paper, CATH groups all these enzymes under the enzyme superfamily, 3.20.20.70 or Aldolase class I; and the PriA enzyme from *Mycobacterium tuberculosis*, despite being comprehensively characterized [30], is still annotated as a HisA enzyme, i.e. only under the EC number 5.3.1.16, and not by both this number and 5.3.1.24, which refers to TrpF or PRA isomerase activity.

In this paper we report that with the use of HMM profiles, and in accordance with their substrate specificities, it is possible to successfully classify sequences of unknown function as HisA or PriA. We identify evolutionary intermediates between HisA and PriA enzyme sub-families within a large metagenome. Selected enzymes, including single amino-acid mutants constructed following evolutionary analyses, as well as a (βα)_6_ truncated enzyme, which may be useful to explore hypotheses related to the evolution of the (βα)_8_-fold, were synthesized and biochemically characterized. Our results demonstrate that substrate specificity within enzyme sub-families, and therefore enzyme promiscuity, can be identified with HMM profiles.

## Methods

### Construction of PriA Hidden Markov Model profiles

The Sequence Alignment and Modeling System program (SAM Version 3.5– T2K) [29] was used to predict the remote intermediates. HMM profiles were built with the w0.5 script [30]. To score the training set of sequences with the HMM the hmmscore program was used. Alignments of the hits were performed with the program align2model [31], and editions and redundancy analyses were performed with Belvu alignment viewer [30]. The best ten hits were aligned to the HMM as above, and the hits of the original entries were removed from the initial training set to avoid re-sampling in the following iteration. The resulting multiple sequence alignment (MSA) was edited, trimmed to the aligning region, and made non redundant at 80 % identity.

### Construction of UniProt database and profile validation

A UniProt database (as of October 2008) consisting of all sequences in the size range of 200 to 300 residues was created. From each scoring run, the sequences with the top 100 E-values were retrieved along with their scores and E-values for further analysis. The data of all rounds were summarized in a matrix describing the E-values of each sequence obtained *per* iteration. Therefore, the entry m_ij_ describes the E-value. The i^th^ is the sequence obtained when evaluated with the profile HMM from the j^th^ iteration.

### Construction of metagenomic database

To generate an initial scoring set, sequences were retrieved from the CAMERA database [32] using the BLAST wizard tool in the webpage http://camera.calit2.net/ (now embedded within the NCBI database). PriA and subHisA sequences from *Actinobacteria*, and HisA sequences from other Eubacteria and Archeae, were BLASTed against all metagenomic ORF peptides (43,240,119 sequences) with default parameters. PriA query was P16250 from *Streptomyces coelicolor* (annotated as both 5.3.1.16 and 5.3.1.24), plus the enzymes annotated as HisA (i.e. only annotated as 5.3.1.16) P9WMM5 from *Mycobacterium tuberculosis* (recently experimentally confirmed as PriA, reference 30), Q8G4S5 from *Bifidobacterium longum*, Q4JW54 from *Corynebacterium jeikenium* (recently confirmed as PriA, reference 27), Q0RFX1 from *Frankia alni*, Q5YYP5 from *Nocardia farcinica*, A8LX58 from *Salinispora arenicola*, A6WCU8 from *Kinecoccocus radiotolerans*, A1R562 from *Arthrobacter aurescens*, A7BD07 from *Actinomyces odontolyticus*, and A5CSK6 from *Clavibacter michiganensis*. One subHisA sequence, O68602 from *Corynebacterium glutamicum*, was also used for BLAST. HisA queries included one Archea: P62356 from *Thermus thermofilus* and six other eubacteria, two Proteobacteria: P10371 from *Escherichia coli* and Q7N8D1 from *Photorhabdus luminiscens*; one cyanobacteria: B0C904 from *Acarychloris marina;* and two firmicutes: O35006 from *Bacillus subtilis* and Q2RGW1 from *Moorella thermoacetica.* The top 100 hits from each run were gathered and repeated sequences above 80 % sequence identity were removed to produce a non-redundant sequence set.

### Phylogenetic analysis of evolutionary intermediates

All MSA were built using MUSCLE within the software SEAVIEW [33]. In order to define the best-fitting model for our data, the program ProtTest [34] was used. The output of this program was used as input for MrBayes, which was run for two million generations [35]. The sequences used, alignment and tree files were deposited in the TreeBASE repository (http://purl.org/phylo/treebase/phylows/study/TB2:S17486) [36].

### Construction of truncated enzyme variants

A library of truncated enzyme variants was constructed by systematically removing one by one the amino acids located at the C-terminus end of the protein, covering a total region of 75 residues. The deletion process was performed by the application of a codon-based oligonucleotide method that we developed, referred to as ‘truncagenesis’. Briefly, the method relies on synthesizing a priming segment of the target oligonucleotide and removing a small fraction of the support, carrying the growing oligonucleotide, every three nucleotides during the synthesis process. The removed fractions are accumulated in a second synthesis column where they are finished with addition of an antisense codon and an appropriate restriction site for cloning purposes, when truncation is targeted at the C-terminus of a protein. Due to technical limitations in the synthesis of long oligonucleotides, coverage of the 75 amino acid targeted region was accomplished by the synthesis of five sets of truncated primers, with each set containing 15 truncated primers. Sequences of the five sets and the primers contained in set1 are shown in Additional file 1: Table S1. Truncated variants were amplified by PCR using the forward primer *Nde*Fw and the corresponding reverse set. The PCR products, as well as the cloning vector, were double digested with the restriction enzymes *Nde*I and *Hind*III, purified, and ligated. Approximately 14,000 colonies were obtained, which represents library coverage of 186X. Plasmids from 32 colonies were isolated and sequenced, validating that the explored region was randomly shortened, as expected.

### Cloning, site-directed mutagenesis and functional analysis of HisA homologs

Sequences retrieved from CAMERA, termed CAM1 and CAM2, and *hisA* from *Acidimicrobium ferrooxidans* genes, were commercially synthesized (Geneart). Codons were optimized for *E. coli* heterologous expression. *priA* from *B. longum* was cloned from genomic DNA generously provided by Frabizio Arigoni (Nestlé Research Center). The full sequence is shown in Additional file 1: Table S2. All genes, including CAM-derived truncated library, were cloned into pASK plasmid [37] using the *Nde*I and *Hin*dIII restriction sites. The pASK plasmid is a version of the commercially available vector pASK_IBA3plus (Iba - Lifesciences), from which the *Nde*I site was deleted by site-directed mutagenesis using the oligonucleotides pASKNdeFor (AAATGATCAATTCAAGGCC) and pASKNdeRev (GCGGATTAGAAAAACAACT). We have previously used pASK for complementation assays involving enzymes with very low promiscuous activities.

In addition, a His-Tag from vector pET15b was cloned using the *Eco*RI and *Hin*dIII restriction sites. The pASK final version contains an *Nde*I site, following the N-terminal His-tag and lacks the C-terminal Strep-tag. CAM mutants’ A81S and A81G were constructed using a site-directed mutagenesis commercial kit (Stratagene) and pASK_CAM1 as template. Sequences of oligonucleotides used are: A81S_F (GTCGAAGTGAGCGGTGGTATCCG) and A81S_Reverse (CGGATACCACCGCTCACTTCGAC) A81G_Forward (GTCGAAGTGGGCGGTGGTATCCG) and A81_R (CGGATACCACCGCCCACTTCGAC). The HisA enzyme from *A. ferrooxidans* was cloned into the pET22b (Novagen) vector using *Nde*I and *Hind*III restriction sites, and PriA from *B. longum*, CAM1_A81G and A81S variants were cloned in the pET28a (Novagen) vector using the *Nde*I and *Xho*I restriction sites. *In vivo* activity complementation assays, as well as *in vitro* steady-state enzyme kinetics, were performed as previously [38], other than pASK derivatives were used and M9 minimal media was supplemented with anhydrous tetracycline at 20 ng/ml (Sigma).

### Construction of 3D structural models

After failed attempts to obtain X-ray crystallographic structures of the proteins investigated, homology models using 2vep structure from *Streptomyces coelicolor* [28] as the initial template were built for CAM1 (261 residues), CAM2 (203 residues) and CAM1_204 (204 residues). Ten thousand decoys were produced with Rosetta 3.4 [39] and clustered for each independent target. The model with the lowest energy of the widest and most populated cluster was chosen as the final structure for each sequence. Protons were placed afterwards, such that an optimal hydrogen-bonding network was achieved, using the WHATIF package [40]. Two additional independent models were produced *in silico*, following the same methodology as before, in which the last 12 residues of the CAM2 sequence were replaced with a randomly generated sequence [41].

## Results

### *Construction of HMM profiles for classification of HisA, PriA* and *subHisA sequences*

Our main goal was to obtain a HMM profile that could be used to classify the HisA/PriA enzyme family into the three known functional groups, each representing one enzyme sub-family: HisA (ProFAR substrate), PriA (ProFAR and PRA substrates) or subHisA (ProFAR substrate). Moreover, in addition to classifying enzyme sub-families, the use of an HMM profile would allow detection of evolutionary intermediates between HisA and PriA enzymes, where divergence is far too large to allow pinpointing key differences at the sequence level. In contrast, the differences between PriA and subHisA sub-families, which show less sequence divergence despite a big functional leap, facilitate the analysis of this process by simpler sequence comparisons (LNG & FBG, unpublished results; [27]). The functional assignment for each enzyme sequence family, i.e. HisA, PriA and subHisA, was done accordingly to the amino acid residues previously identified as specific for each sub-family, as describe in the Background section and references therein (see also Additional file 1: Figure S1).

The seed sequence used for the initial HMM profile was that of the well-characterized PriA enzyme from *S. coelicolor*, referred to as PriA_Scoe [26, 28, 38, 42]. HisA sequences, from *Bacillus subtilis* and relatives, were also used for construction of HMM profiles. Probably due to the larger heterogeneity of HisA enzymes in the databases, results obtained using PriA gave better resolution early on during these analyses (data not shown). Thus, the PriA HMM profile was further developed, and subsequent PriA models incorporated new sequences in each iteration, with redundancy being eliminated at 80 % in each step. For instance, “Q5YYP5” from *Nocardia farcinica* and “A1T8W5” from *Mycobacterium vanbaaleni*, which were present in the model of iteration 2, were removed, as their similarity is above this threshold.

The sequences that were included at each iteration, as well as those that were removed are shown in Table 1. The top ten hits obtained after all iterations were aligned to the model as part of the MSA that was used to build the resulting HMM. Therefore, every iteration involved: (*i*) construction of a HMM profile, which was used to score a training set; (*ii*) subtraction of the top scoring sequences from the training set; (*iii*) alignment of these sequences with the HMM and the previous sequences; and (*iv*) editing of the MSA to build the model for the next iteration. This process yielded one HMM per iteration until it was stopped at the seventh iteration, as no further actinobacterial sequences were retrieved.

**Table 1:** Sequences included at each HMM profile

	HMM profile							
UniProt entry^1^	Organisms	Iter2	Iter3	Iter4	Iter5	Iter6	Iter7	RF
P16250	***Streptomyces coelicolor***	X	X	X	X	X	X	X
Q47QS4	***Thermobifida fusca***	X	X	X	X	X	X	X
A6WCU8	***Kinecococcus radiotolerans***	X	X	X	X	X	X	X
A0LTS5	***Acidothermus cellulolyticus***	X	X	X	X	X	X	X
Q5YYP5	***Nocardia farcinica***	X						
A1T8W5	***Mycobacterium vanbaaleni***	X						
A4FLL9	***Saccharopolyspora erythrea***		X	X	X	X	X	X
B1MBX5	***Mycobacterium abscessus***		X	X	X	X	X	X
Q9CC56	***Mycobacterium leprae***		X	X	X	X	X	X
A0JUZ7	***Arthrobacter*** **sp.**			X	X	X	X	X
B1VDA6	***Rhodococcus erythroplis***			X	X	X	X	X
A8LX58	***Salinispora arenicola***			X	X	X	X	X
A1SL57	***Nocardioides*** **sp.**			X	X	X	X	X
Q4JW54	***Corynebacterium jeikeium***			X	X	X	X	X
Q2J8L2	***Frankia*** **sp.**			X	X	X	X	X
A4AHK5	***marine actinobacteria***				X	X	X	X
C5C9W9	***Micrococcus luteus***				X	X	X	X
J2ZMD0	***Actinomyces naeslundi***				X	X	X	X
A5CSK6	***Clavibacter michiganensis***				X	X	X	X
A3TLY4	***Janibacter*** **sp.**				X	X	X	X
Q6AE15	***Leifsonia xyli***				X	X	X	X
Q8G4S5	***Bifidobacterium longum***					X	X	X
Q6A8L1	***Propionibacterium acnes***					X	X	X
A7BD07	***Actynomyces odontolyticus***					X	X	
Q7N8D1	*Photorhabdus luminescens*					X	X	
B1I557	*Desulforudis audaxviator*						X	
Q2RGW1	*Moorella thermoacetica*						X	
A5CZ74	*Pelotomaculum thermoprpionicum*						X	
A4J708	*Desulfotomaculum reducens*						X	
B1SQW0	*Geobacillus* sp.						X	
B0TDM8	*Heliobacterium modesticaldum*						X	
A4ISR3	*Geobacillus thermodentrificans*						X	
Q2B721	*Bacillus* sp.						X	
P74561	*Synechocystis* sp.						X	
A0YW53	*Lyngbya* sp.						X	
O35006	*Bacillus subtilis*						X	

^1^Accession number into Uniprot. The PriA homologs from the class *Actinobacteria* are highlighted in bold. HisA homologues from *Firmicutes*, *Proteobacteria* and *Cyanobacteria* are not shadowed. The sequences included in each HMM profile are shown as X. Iter# (from 2 till 7) refers to the number of iteration made to construct the HMM. RF stands for ReFined model obtained after iteration 6

**Fig. 2:**
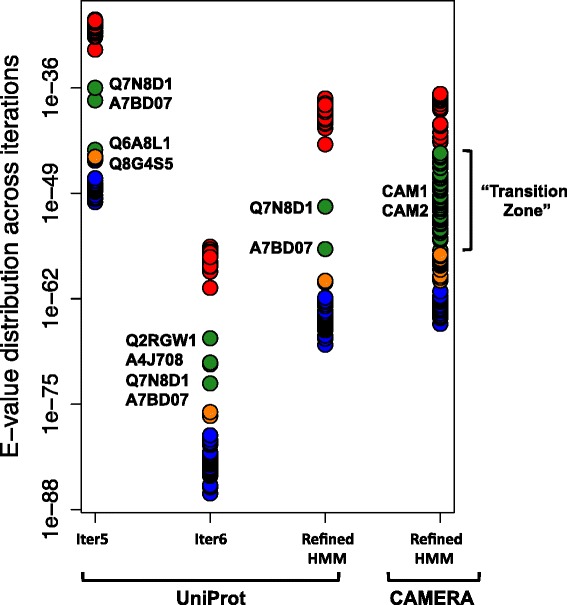
Performance of HMM profiles against UniProt and CAMERA databases. HisA, PriA, subHisA and ‘Transition Zone’ sequences are shown as red, blue, orange and green dots, respectively. UniProt codes corresponding to sequences removed to construct the HMM refined profile are indicated. The functionally characterized evolutionary intermediates from CAMERA database are marked as CAM1 and CAM2

A total of seven HMM profiles were obtained and evaluated. Optimal profiles were defined as PriA sequences with low variance between the assigned E-values and with significantly lower E-values for PriA than for HisA. Based on these criteria, profiles 5 and 6 were the only ones that could differentiate between the HisA and PriA sub-families. For the sake of clarity, only results from profiles 5 and 6 are shown in Fig. 2. However, none of the profiles could separate subHisA (orange dots) from the PriA group (blue dots). In addition, using profiles 5 and 6, some PriA sequences coming from deep-rooted *Actinobacteria*, as well as some HisA sequences (red dots) coming from organisms belonging to the Firmicutes or Gram-positive with low (G + C) content bacteria (black dots), could not be classified. The region that includes these outlying sequences represents the transition between these enzyme groups, labeled as transition zone (TZ) in Fig. 2.

To improve the HMM profile, a refined profile, constructed from profile 6, was obtained. This was done after arbitrarily removing the outlying sequences “A4J708” and “Q2RGW1” of the Firmicutes *Desulfotomaculum reducens* and *Moorella thermoacetica*, as well as “Q7N8D1” and “A7BD07” from the enteric bacterium *Photorhabdus luminescens* and the actinobacterium *Actinomyces odontolyticus*, respectively (Fig. 2). These outlying sequences, in particular the latter, may provide interesting candidates for future functional characterization with very unique evolutionary histories, but for the purpose of HMM construction they were removed, as previously advised [17]. This step leads to a more homogeneous sequence sub-sample and increases the resolution of the new model. Indeed, this refined PriA model no longer misclassifies Firmicutes sequences as PriA enzymes, and instead correctly classifies them as HisA enzymes.

Use of the refined profile allowed us to classify UniProt HisA and PriA sequences, from which 58 selected non-redundant sequences were retrieved. These sequences were successfully grouped, with a difference of nineteen orders of magnitude, into HisA (E-values of 5.55E-44 and smaller) and PriA (E-values between 1E-69 and 6.8E-57). In addition, the PriA sequences were slightly separated into two sub-groups. The first sub-group corresponds to dual-substrate PriA enzymes from actinobacterial organisms (E-values between 1E-69 and 5.07E-61), while the second group corresponds to subHisA enzymes from the previously identified sub-clade of the genus *Corynebacterium* (between 6.8E-57 and 5.55E-44). The distribution of these enzymes was 50 % HisA, 41.37 % PriA, 5.17 % subHisA. However, only 3.4 % was assigned to the TZ sub-set (green dots).

### Identification of metagenomic evolutionary intermediates between HisA and PriA

Once our HMM refined profile was validated using UniProt, it was used to search for promiscuous evolutionary intermediates in the large and diverse metagenomic database CAMERA. At the time when these sequence searches were done, this database had been shown to contain higher diversity, useful for sequence bioprospecting [32, 43]. To define the E-value threshold between PriA and subHisA groups, the highest E-value of the PriA group and the smallest E-value of the subHisA group obtained from UniProt were selected. The former was subtracted from the latter and the result was divided by two, giving a value that was added to the highest E-value found for PriA. This is equivalent to subtracting the same value from the lowest E-value found in HisA. The result of this calculation gives the threshold E-value for classification to functional groups. Thus, sequences with lower E-values were considered PriA, and sequences with higher E-values were considered subHisA. The same procedure was applied to calculate the E-value threshold between subHisA or HisA and the functionally ambiguous enzymes present in the TZ.

Use of the refined profile allowed us to classify the previously built metagenomic database, consisting of 147 non-redundant sequences. These sequences were grouped into the three known sub-families with a distribution of 23.1 % HisA, 27.2 % PriA and 9.5 % subHisA. Moreover, the TZ group now included up to 40 % of the sequences, increasing the likelihood of finding evolutionary intermediates. Unlike the E-value distribution of the UniProt sequences, the distribution of E-values of CAMERA sequences shows a continuum that covers the E-value distance between HisA and PriA, *i.e.* the entire TZ (Fig. 2, green dots).

**Fig. 3:**
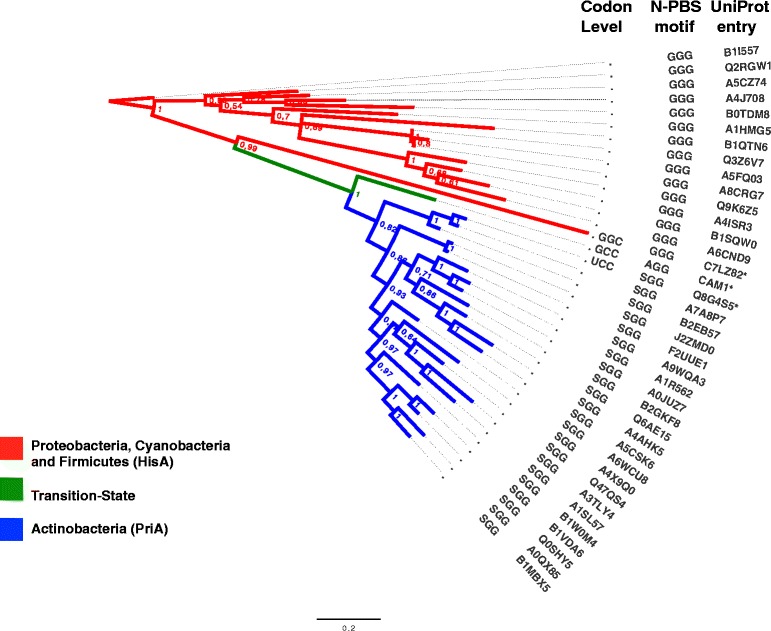
Phylogenetic analysis of HisA / PriA evolutionary intermediates. A Bayesian phylogenic tree of HisA homologs is shown. Minimum branch support is 0.7. HisA, PriA and intermediate sequences, as CAM1, are grouped in different clades, labeled in red, blue and green, respectively. The key enzymes HisA_Afer (C7LZ82) and PriA_Blon (Q8G4S5) are also shown. The evolutionary intermediate, reflected at the codon level in the N-PBS motif, is shown. Functionally analyzed proteins, and their first N-PBS amino acid codon usage, are marked with an asterisk

As previously stated, and also shown in Fig. 3 and Additional file 1: Figure S1, HisA and PriA sequences have a glycine or a serine residue, respectively, in position 81. However, none of the presumed subHisA sequences (orange dots in CAMERA) has the threonine in this position, which is the distinctive feature of this sub-family [27]. This may be due to the fact that CAMERA is based on marine samples, and species belonging to the genus *Corynebacterium* may not be present, at least not prominently, in marine environments. This observation makes it unlikely that these sequences are in fact subHisA sequences, and may well be divergent PriA homologs from uncultivated distantly related taxa. Lacking of further data providing insights into the nature of these sequences, we found it unwise to further characterize these enzymes functionally.

The TZ group includes an additional 60 sequences (Fig. 2, **green dots**), which have either glycine or serine in position 81, making them closer to either HisA or PriA, respectively. Interestingly, exceptions to this observation were found in two sequences that have an alanine in this position, which we called CAM1 and CAM2. Alanine is one of the possible transitional states from glycine (HisA) to serine (PriA) at the codon level. The mutational path GGC (Gly) → GCC (Ala) → UCC (Ser) modifies the N-terminal PBS, from GlyGlyGly to AlaGlyGly and finally into SerGlyGly (Fig. 3). As the sequences of CAM1 and CAM2 suggest that they are evolutionary intermediates, these putative enzymes were investigated further by means of sequence, structural and functional analyses.

At the sequence level, CAM1 and CAM2 were found to be almost identical, other than their C-terminal ends. As shown in the MSA of Additional file 1: Figure S1, CAM1 has 261 amino acids, which is the average length of both HisA and PriA enzymes. In contrast, CAM2 has only 203 amino acids, of which all except the last 12 residues are identical to CAM1 (see also Fig. 5). Thus, a phylogenetic analysis using only the complete sequence of CAM1, and not CAM2, was performed. This analysis also included selected sequences obtained from public databases, as retrieved at time of submission, using standard sequence similarity searches. The resulting phylogenetic tree is shown in Fig. 3.

The HisA clade (red branches) encompasses HisA enzymes from Proteobacteria, Firmicutes and Cyanaobacteria, and it serves as the root. In the transition between HisA and PriA, we found the HisA enzyme from *Acidimicrobium ferrooxidans* (termed HisA_Afer), an organism that belongs to the high (G + C) content Gram-positive bacteria, but a different order than actinobacteria. Moreover, the outgroup of the PriA clade (green branch) is indeed CAM1. As expected, the enzyme “Q8G4S5” from *Bifidobacterium longum* (termed PriA_Blon), an outgroup of the actinobacteria belonging to the order Bifidobacteriales, as well as the actinobacterial deep-rooted genus *Actinomyces*, appear as sister taxa of CAM1.

### Functional analysis of evolutionary intermediates

We then decided to experimentally investigate the specificities, towards PRA (TrpF activity) and ProFAR (HisA activity), of HisA_Afer and PriA_Blon, as well as CAM1 and the truncated version CAM2 (Table 2). All genes were cloned into suitable plasmids for complementation, protein over-expression and purification procedures. However, despite our multiple attempts to express and purify CAM2, this hypothetical protein could not be overexpressed to a level where purification could be attained. Nevertheless, *in vivo* complementation of a *hisA* minus *E. coli* mutant, termed HfrG6 [44], showed that HisA_Afer, PriA_Blon, and the putative evolutionary intermediates CAM1 and CAM2, all have ProFAR isomerase or HisA activity. However, cells complemented with CAM2 showed significantly reduced growth. Moreover, based on similar *in vivo* complementation experiments, but using a *trpF* minus *E. coli* mutant termed FBG-W_f_ [28], PRA isomerase activity could only be found for PriA_Blon and CAM1, but not for CAM2 or HisA_Afer (Additional file 1: Figure S2).

**Table 2:** *In vivo* and *in vitro* characterization of selected HisA/PriA homologs and mutants

Enzymes	*In vivo* activity		*In vitro* activity^a^					
			ProFAR isomerase (HisA)			PRA isomerase (TrpF)		
	HisA	TrpF	*K*_*M*_ μM	*k*_*cat*_ s^−1^	*k*_*ca*t_/*K*_*M*_ s^−1^ μM^−1^	*K*_*M*_ μM	*k*_*cat*_ s^−1^	*k*_*cat*_/*K*_*M*_ s^−1^ μM^−1^
HisA_Afer	+	-	1.1 ± 0.2	0.05 ± 0.001	0.045	n.d.	n.d.	n.d.
PriA_Blon	+	+	2.7 ± 0.5	0.4 ± 0.1	0.1	6.1 ± 0.1	2.1 ± 0.5	0.3
CAM1	+	+	1.7 ± 0.1	0.3 ± 0.03	0.2	40 ± 7	3.5 ± 0.04	0.09
CAM1_A81G	+	+	1.7 ± 0.2	0.1 ± 0.01	0.06	32.2 ± 1.7	1.9 ± 0.1	0.06
CAM1_A81S	+	+	4.0 ± 0.9	0.2 ± 0.03	0.04	23.5 ± 6.5	0.5 ± 0.1	0.02
CAM2	+	-	n.d.	n.d.	n.d.	n.d.	n.d.	n.d.

^a^Each data point comes from at least three independent determinations using freshly purified enzyme. n.d., activity not detected

**Fig. 4:**
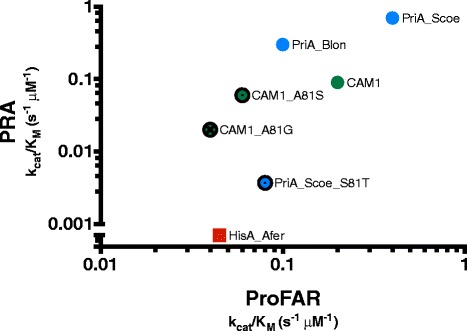
Comparison of steady-state enzyme kinetics. A graphical comparison of the catalytic efficiencies (*k*_*cat*_/*K*_M_) of wild type and mutant enzymes that were characterized is shown. Values for ProFAR (*x* axis) and PRA (*y* axis) isomerase activities, expressed as log10, are compared. Data from HisA_Afer (red square), PriA_Blon (blue circle), CAM1 (green circle), CAM1_A81S (green circle with a black border and inner black circle) and CAM1_A81G (green circle with a black border and inner black cross, this study), as well as from PriA_Scoe (blue circle) and the Ser81Thr mutant of PriA from *S. coelicolor*, labeled as PriA_Scoe (blue circle with a black border and inner black circle) (data obtained from [38]), is included

The aforementioned *in vivo* results were confirmed by *in vitro* steady-state enzyme kinetics, which led to *k*_*cat*_ and *K*_M_ parameters for all enzymes analyzed, other than for CAM2, which could not be purified. A decreased ProFAR isomerase activity for HisA_Afer was detected, which may be due to optimal pH 2.2 growth conditions of the organism from which this enzyme was obtained [45]. No differences in activity, however, could be detected when the enzyme assays were done at different pH values, ranging from 2.6 till 7.5 (Additional file 1: Figure S3). It was also noted that CAM1 has a decreased PRA isomerase activity, consistent with the proposed nature of a promiscuous evolutionary intermediate (Table 2 and Fig. 4). Indeed, inspection of the kinetic parameters of all these enzymes shows that CAM1 has a very poor *K*_M_ value for PRA (40 ± 7 μM), at least one order of magnitude higher than that found for PriA_Blon (6.1 ± 0.1 μM). This observation implies that CAM1’s low TrpF or PRA isomerase activity relates to its inability to properly bind the substrate PRA.

Altogether, these results imply (*i*) the existence of HisA enzymes able to bind PRA promiscuously, or early PriA enzymes with dual-substrate specificity, in the root of *Actinobacteria*; (*ii*) that CAM1 has the potential for being a HisA to PriA evolutionary intermediate, as predicted by our HMM refined profile and suggested by its nascent PRA isomerase activity; and (*iii*) despite CAM2 having ProFAR isomerase activity *in vivo*, as suggested by its truncated nature and the difficulties encountered during its characterization, this protein may be an artifact derived from sequence misannotation, and not a naturally occurring (βα)_6_-barrel .

### Mutagenesis and structural analysis of CAM1 and CAM2 variants

Serine 81, located at the N-terminal PBS motif of PriA, is part of the active site and its specific role in the binding of PRA (TrpF substrate), and not ProFAR (HisA substrate), has been suggested by enzyme kinetics and X-ray crystallographic structural analysis [38, 46]. CAM1, as previously mentioned, has an alanine in this position. Site-directed mutagenesis of this residue into glycine (HisA direction) and serine (PriA direction) was done. As can be observed in Table 2 and Fig. 4, the mutants CAM1_A81G and CAM1_A81S retain both activities. CAM1_A81G presents similar ProFAR and PRA isomerase activities to those found for CAM1, whereas CAM1_A81S activities were only reduced 8-fold, i.e. not even an order of magnitude. This observation shows that this position in CAM1 can be mutated without major consequences upon catalytic efficiency, which contrasts with the dramatic effect that mutation of Ser81 in PriA, even into a highly similar amino acid such as threonine, has upon its isomerase activities (between one and two orders of magnitude; Fig. 4).

**Fig. 5:**
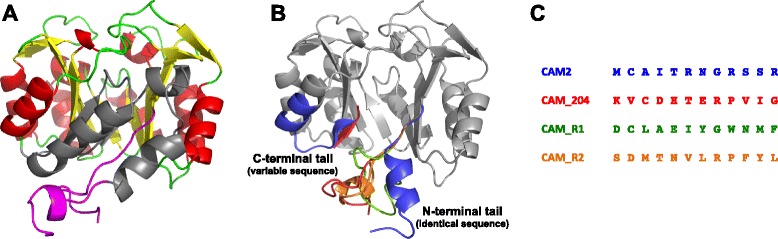
Structural analysis of CAM1 and CAM2. **a**. Structural homology model of CAM1, corresponding to a full (βα)_8_-barrel, and CAM2. The (βα)_2_ subunit missing in CAM2 is shown in gray. N and C-terminal tails are shown in magenta, loops, alpha helix and beta sheet are marked in green, red and yellow, respectively. **b**. Structural superposition of CAM2 (gray backbone), CAM2_204, CAM2_R1 and CAM2_R2, where N and C-terminal tails are shown in blue, red, green and orange, respectively. **c**. Sequence alignment of the 12-amino acid C-terminal variable region of CAM2, CAM2_204 (equivalent to CAM1), CAM2_R1 and CAM2_R2. The pairwise RMSD between CAM1 and CAM2, CAM2 and CAM2_R1, CAM2 and CAM2_R2, and CAM2 and CAM2_204 was 1.73 Å, 1.91 Å, 1.41 Å, 1.73 Å, respectively

Crystallographic structural characterization was not possible; therefore, we built homology-based structural predictions for CAM1 and CAM2. As expected, CAM1 is predicted to fold as a complete (βα)_8_ barrel with two unstructured tails of ~10 amino acids at the N and C terminal regions. In contrast, CAM2 is predicted to lack the last two units of the (βα)_8_ barrel, leading to a putative (βα)_6_-barrel. The model of CAM2 shows two unstructured tails, that of the N-terminal region identical to CAM1, and a 12 amino acid C-terminal tail different from CAM1 (Fig. 5). Given the different enzymatic activities found in CAM1 and CAM2, and starting from the sequence of CAM1, we designed a library of truncated variants. In this library, CAM1 was truncated by one amino acid at a time, until reaching 186 amino acids, with the aim of isolating (βα)_6_-barrels with activity.

A library of 14,000 clones was obtained by PCR assembly and cloning into pASK vector, as described in the Materials and Methods. The CAM1 truncated library was screened for ProFAR isomerase activity by complementation using the *E. coli hisA*^*−*^ strain HfrG6. Colonies that could grow in minimal medium were pooled together and used for screening of PRA isomerase activity in the *E. coli trpF*^*−*^ strain FBG-W_f_, as before. Approximately 20 positive clones were isolated and their sequences obtained and analyzed. The results show that only CAM1 variants with lengths between 251 and 260 amino acids could be recovered. According to our structural model, the last 10 amino acids correspond to a tail with no secondary structure (Fig. 5A). Thus, all CAM1 truncated variants that are active, most likely fold as a complete (βα)_**8**_ barrels.

We sequenced CAM1 variants immediately after screening for the ProFAR isomerase activity. Not even variants with the same length to CAM2, but with a C-terminal region identical to CAM1 (CAM2_204) or even slighter longer (CAM2_215), had ProFAR isomerase activity. Moreover, deletion of the non-conserved C-terminal end of CAM2, rendering a protein with 186 amino acids (CAM2_186), abolished the incipient ProFAR isomerase activity found in CAM2 (Additional file 1: Figure S2). All together, these analyses support the idea that CAM2 is an artifact, probably coming from misannotation, and not a naturally occurring protein. Nevertheless, CAM2 could provide an interesting protein model to investigate the evolution of the (βα)_8_ fold from smaller subunits.

To further investigate CAM2, we modeled the structure of CAM2_204. As can be observed in Fig. 5b, the structure of the C-terminal regions of both CAM2 and CAMs_204 have high similarity. Although these observations are based on computational models, it was interesting to find differences between these two enzymes at the N-terminal region, which corresponds to the first 15 amino acids of this tail. CAM2 can condense a small alpha helix at this end, while CAM1 and CAM2_204 are predicted to be unable to do so. To test if the 12-residue sequence found at the C-terminal of CAM2 is responsible for the structural variation at its N-terminal region, two additional independent structural models were produced (Fig. 5b & c). The last 12 residues of the sequence of CAM2 were replaced with a randomly generated sequence. The first variant had an amino acid distribution that mimicked that of CAM1, while the second variant had an amino acid distribution that mimicked that observed in nature. These models were termed CAM2_R1 and CAM2_R2, respectively, and no alpha helix condensation could be found at their N-terminal tails (Fig. 5b).

## Discussion

Our report represents an effort to classify enzyme sub-families with different substrate specificities, aiming to predict enzyme promiscuity in non-annotated databases. For this purpose, we used HMM profiles and (βα)_8_ isomerases involved in L-histidine and L-tryptophan biosynthesis, two ancestral pathways, for proof-of-concept. The use of HMM profiles allowed us to distinguish between the closely related enzyme sub-families HisA (mono-substrate) and PriA (dual-substrate), but not the evolutionarily more closely related subHisA (mono-substrate). After validation of our PriA HMM profile we classified a subset of HisA homologs contained in the CAMERA metagenomic database. Interestingly, discrete groups were no longer obtained; on the contrary, a continuum of E-values was found (transition zone). This seems to correlate with a higher degree of diversity contained within non-cultivated microorganisms, as previously widely noted [47, 48].

By means of MSA, we detected two evolutionarily intermediate sequences, CAM1 and CAM2, with a unique PBS sequence motif. The mutational path from glycine to serine requires a two-nucleotide substitution, with alanine being one of the plausible transition amino acids. Although such amino acid transition has been previously observed in the laboratory after directed evolution experiments [49], to the extent of our knowledge, it is rare to find homologous enzymes that enable the study of a natural mutational path. Thus, the existence of such a scenario, represented here by evolution of PriA from HisA via CAM, support the idea that CAM1 and CAM2 can be assumed to be enzymes representing possible evolutionary trajectories. Along these lines, it would be interesting to take into account residues unrelated to the N-PBS but that directly mediate substrate specificity, such as the loop-embedded Arg19 and Trp145 involved in the metamorphosis of the active site of PriA [29, 30].

Dedicated phylogenetic analysis of HisA, CAM and PriA enzymes, placed CAM1 as the outgroup of PriA sub-family, between the genera *Acidimicrobium* and *Bifidobacterium*. In accordance with the bacterial tree of life [50–52] *Acidimicrobium* species, a group of early divergent Gram positive with high (G + C) content bacteria, is the outgroup of *Bifidobacterium*. Moreover, the genus *Bifidobacterium* is the outgroup of the order *Actinomycetales*, which includes species where PriA has been found as a response to the lack of a *trpF* gene [26, 27, 46]. In agreement with this, HisA_Afer was shown to have specificity towards ProFAR, while PriA_Blon can accept both PRA and ProFAR substrates. These observations suggest that evolution of PriA from HisA could have occurred in the divergence events that gave rise to the Gram positive with high (G + C) content bacterial group, dated to 2 billion years ago [50].

Mutation of residue 81 (glycine in HisA and serine in PriA) could be tolerated by CAM1, which contrasts with the effect of mutating this residue in PriA from *S. coelicolor* (Fig. 4) [28, 38]. The reason why CAM1 could withstand mutations in this key catalytic residue is not understood. It implies, however, an increased robustness, a characteristic that could be intrinsic to evolutionary intermediates, in this case between the HisA and PriA sub-families. Given recent reports, it is tempting to speculate that this observation has to do with the role of epistasis during early enzyme evolution, which involves promiscuous enzyme states [8–11]. This could be exploited as an attribute for detection and annotation of enzyme promiscuity from sequence data using HMM profiles.

Given the nature of neutral mutations, the development of systematic approaches to detect enzyme promiscuity at the sequence level has been hindered [2]. Based on molecular signatures obtained by means of graph kernel support vector machines, it has been suggested previously that it is possible to make predictions with an accuracy of 85 % [53, 54]. More recently, random forests have been used to predict enzyme substrate-specific residues, which are intimately related to enzyme promiscuity [55]. However, although these methods claim to predict function at the fourth level of the Enzyme Classification number, a situation that remains to be experimentally validated, incorrect annotation in current databases, incorrect training of the method, domain fusions, and bias in the knowledge of functional residues, among other factors, are likely to hamper their efficiency. Our report expands on this repertoire of approaches, with the advantage of being computationally simpler, and possibly also more sensitive as it is based in HMMs.

Two plausible scenarios have been hypothesized for the evolution of the (βα)_**8**_-fold. First, based on the symmetry seen in HisA and its remote homolog HisF, whose substrates/products are also symmetrical, duplication and subsequent fusion of (βα)_4_ half barrels has been put forward [19]. This hypothesis has been supported experimentally by reconstitution of complete (βα)_**8**_ barrels from two (βα)_4_ halves [20, 56]. Second, an ‘asymmetrical’ pathway, which considers two, three or more duplication and fusion events, proposes that the (βα)_**8**_ barrel could have originated from (βα)_2_ and (βα)_3_ subunits [23, 57]. Indeed, it has been shown experimentally that (βα)_6_ barrels are viable, and thus they may also provide intermediaries in the course of evolution and appearance of the (βα)_8_ [22, 24].

Isolation of a functional HisA-like (βα)_**6**_ barrel in this study suggests that the latter ‘non-symmetrical’ hypothesis may also be viable for symmetrical proteins. Unfortunately, despite several efforts, we could not obtain crystallographic structural data for either CAM1 or CAM2. This observation, together with our inability to isolate (βα)_**6**_ active proteins after screening of a CAM1 truncated library, suggests that CAM2 is not a naturally occurring protein, but rather a fictitious protein arising from misannotation of metagenomic DNA. Nevertheless, our homology-based structural modeling supports the idea that CAM2 maintains some activity because its amino acid sequence at the C-terminal end allows a stable secondary structure. This predicted structural feature may stabilize the (βα)_**6**_ fold, and thus its active site, analogous to cases in which remote functional mutations have been found [14, 15]. In the absence of the C-terminal PBS, needed to bind the symmetrical ProFAR substrate, an interesting possibility along these lines would be that CAM2 undergoes dimerization and domain swapping reconstitute its active site.

## Conclusion

As expected, reconstruction of the evolution of PriA from HisA, using HMM profiles and phylogenetics, suggest functional shifts involving intragenic epistatic mutations. These mutations seem to be compatible with a stability-activity tradeoff that allows a broader exploration of sequence space by nature. Indeed, as the evolutionary intermediates identified here behave as promiscuous enzymes, a key evolutionary raw material, intragenic epistasis is confirmed as a mechanism driving functional shifts. The use of HMM provides a convenient approach for gaining insights into these evolutionary processes, as was shown here for the evolution of PriA from HisA, but not for subHisA from PriA, as the latter are evolutionarily closely related and thus highly similar. Isolation of a (βα)_**6**_ protein with HisA activity provides further evidence for the hypothesis that extant (βα)_**8**_ may have evolved not only from symmetrical subunits.

## Additional file

Additional file 1: Figure S1. Sequence alignment of functionally characterized proteins. **Figure S2.** *E. coli trpF* ^*−*^ *and hisA* ^*−*^ complementation assays with selected proteins. **Figure S3.** Catalytic activity pH dependence of HisA_Afer. **Table S1.** Oligonucleotides used to construct CAM1 truncated variants. **Table S2.** Protein sequence of synthetic genes.

## References

[1] Khersonsky O, Roodveldt C, Tawfik DS (2006). Enzyme promiscuity: evolutionary and mechanistic aspects. Curr Opin Chem Biol.

[2] Khersonsky O, Tawfik DS (2010). Enzyme promiscuity: a mechanistic and evolutionary perspective. Annu Rev Biochem.

[3] Notebaart RA, Szappanos B, Kintses B, Pal F, Gyorkei A, Bogos B (2014). Network-level architecture and the evolutionary potential of underground metabolism. Proc Natl Acad Sci U S A.

[4] Jensen RA (1976). Enzyme recruitment in evolution of new function. Annu Rev Microbiol.

[5] Soskine M, Tawfik DS (2010). Mutational effects and the evolution of new protein functions. Nat Rev Genet.

[6] Verdel-Aranda K, López-Cortina ST, Hodgson DA, Barona-Gómez F. Molecular annotation of ketol-acid reductoisomerases from Streptomyces reveals a novel amino acid biosynthesis interlock mediated by enzyme promiscuity. Microb Biotechnol. 2015;8(2):239-52. 10.1111/1751-7915.12175 PMC4353338 25296650

[7] Bloom JD, Romero PA, Lu Z, Arnold FH. Neutral genetic drift can alter promiscuous protein functions, potentially aiding functional evolution. Biol Direct. 2007; 28;2:17. 10.1186/1745-6150-2-17 PMC1914045 17598905

[8] Garcia-Seisdedos H, Ibarra-Molero B, Sanchez-Ruiz JM. Probing the mutational interplay between primary and promiscuous protein functions: a computational-experimental approach. PLoS Comput Biol. 2012;8(6):e1002558. 10.1371/journal.pcbi.1002558 PMC3375227 22719242

[9] Huang RQ, Hippauf F, Rohrbeck D, Haustein M, Wenke K, Feike J (2012). Enzyme functional evolution through improved catalysis of ancestrally nonpreferred substrates. Proc Natl Acad Sci U S A.

[10] Sanchez-Ruiz JM (2012). On promiscuity, changing environments and the possibility of replaying the molecular tape of life. Biochem J.

[11] Parera M, Martinez MA (2014). Strong Epistatic Interactions within a Single Protein. Mol Biol Evol.

[12] Zhang W, Dourado DF, Fernandes PA, Ramos MJ, Mannervik B (2012). Multidimensional epistasis and fitness landscapes in enzyme evolution. Biochem J.

[13] Khanal A, Yu McLoughlin S, Kershner JP, Copley SD: Differential effects of a mutation on the normal and promiscuous activities of orthologs: implications for natural and directed evolution. Mol Biol Evol. 2015;32(1):100-8. 10.1093/molbev/msu271 PMC4271523 25246702

[14] Boucher JI, Jacobowitz JR, Beckett BC, Classen S, Theobald DL. An atomic-resolution view of neofunctionalization in the evolution of apicomplexan lactate dehydrogenases. Elife. 2014; 25;3. 10.7554/eLife.02304 PMC4109310 24966208

[15] Ochoa-Leyva A, Barona-Gomez F, Saab-Rincon G, Verdel-Aranda K, Sanchez F, Soberon X (2011). Exploring the Structure-Function Loop Adaptability of a (beta/alpha) (8)-Barrel Enzyme through Loop Swapping and Hinge Variability. J Mol Biol.

[16] Finn RD, Mistry J, Tate J, Coggill P, Heger A, Pollington JE (2010). The Pfam protein families database. Nucleic Acids Res.

[17] Eddy SR (2004). What is a hidden Markov model?. Nat Biotechnol.

[18] Farias-Rico JA, Schmidt S, Hocker B (2014). Evolutionary relationship of two ancient protein superfolds. Nat Chem Biol.

[19] Lang D, Thoma R, Henn-Sax M, Sterner R, Wilmanns M (2000). Structural evidence for evolution of the beta/alpha barrel scaffold by gene duplication and fusion. Science.

[20] Hocker B, Beismann-Driemeyer S, Hettwer S, Lustig A, Sterner R (2001). Dissection of a (betaalpha)8-barrel enzyme into two folded halves. Nat Struct Biol.

[21] Hocker B, Claren J, Sterner R (2004). Mimicking enzyme evolution by generating new (betaalpha)8-barrels from (betaalpha)4-half-barrels. Proc Natl Acad Sci U S A.

[22] Patrick WM, Blackburn JM (2005). In vitro selection and characterization of a stable subdomain of phosphoribosylanthranilate isomerase. FEBS J.

[23] Richter M, Bosnali M, Carstensen L, Seitz T, Durchschlag H, Blanquart S (2010). Computational and experimental evidence for the evolution of a (beta alpha)8-barrel protein from an ancestral quarter-barrel stabilised by disulfide bonds. J Mol Biol.

[24] Setiyaputra S, Mackay JP, Patrick WM (2011). The structure of a truncated phosphoribosylanthranilate isomerase suggests a unified model for evolution of the (betaalpha)8 barrel fold. J Mol Biol.

[25] List F, Sterner R, Wilmanns M (2011). Related (betaalpha)8-barrel proteins in histidine and tryptophan biosynthesis: a paradigm to study enzyme evolution. Chembiochem.

[26] Barona-Gomez F, Hodgson DA (2003). Occurrence of a putative ancient-like isomerase involved in histidine and tryptophan biosynthesis. EMBO Rep.

[27] Noda-Garcia L, Camacho-Zarco AR, Medina-Ruiz S, Gaytan P, Carrillo-Tripp M, Fulop V (2013). Evolution of substrate specificity in a recipient’s enzyme following horizontal gene transfer. Mol Biol Evol.

[28] Wright H, Noda-Garcia L, Ochoa-Leyva A, Hodgson DA, Fulop V, Barona-Gomez F (2008). The structure/function relationship of a dual-substrate (betaalpha)8-isomerase. Biochem Biophys Res Commun.

[29] Hughey R, Krogh A: SAM: Sequence alignment and modeling software system. In*.* Santa Cruz, CA, USA: University of California; 1995.

[30] Sonnhammer EL, Hollich V (2005). Scoredist: a simple and robust protein sequence distance estimator. BMC Bioinformatics.

[31] Karplus K, Barrett C, Hughey R (1998). Hidden Markov models for detecting remote protein homologies. Bioinformatics.

[32] Seshadri R, Kravitz SA, Smarr L, Gilna P, Frazier M (2007). CAMERA: a community resource for metagenomics. PLoS Biol.

[33] Gouy M, Guindon S, Gascuel O (2010). SeaView version 4: A multiplatform graphical user interface for sequence alignment and phylogenetic tree building. Mol Biol Evol.

[34] Abascal F, Zardoya R, Posada D (2005). ProtTest: selection of best-fit models of protein evolution. Bioinformatics.

[35] Ronquist F, Huelsenbeck JP (2003). MrBayes 3: Bayesian phylogenetic inference under mixed models. Bioinformatics.

[36] Shan HY, Herbert KG, Piel WH, Shasha D, Wang JTL (2002). A structure-based search engine for phylogenetic databases.

[37] Adams NE, Thiaville JJ, Proestos J, Juarez-Vazquez AL, McCoy AJ, Barona-Gomez F (2014). Promiscuous and adaptable enzymes fill “holes” in the tetrahydrofolate pathway in Chlamydia species. mBio.

[38] Noda-Garcia L, Camacho-Zarco AR, Verdel-Aranda K, Wright H, Soberon X, Fulop V (2010). Identification and analysis of residues contained on beta – > alpha loops of the dual-substrate (beta alpha)8 phosphoribosyl isomerase A specific for its phosphoribosyl anthranilate isomerase activity. Protein Sci.

[39] Leaver-Fay A, Tyka M, Lewis SM, Lange OF, Thompson J, Jacak R (2011). ROSETTA3: an object-oriented software suite for the simulation and design of macromolecules. Methods Enzymol.

[40] Vriend G (1990). WHAT IF: a molecular modeling and drug design program. J Mol Graph.

[41] Artimo P, Jonnalagedda M, Arnold K, Baratin D, Csardi G, de Castro E (2012). ExPASy: SIB bioinformatics resource portal. Nucleic Acids Res.

[42] Kuper J, Doenges C, Wilmanns M (2005). Two-fold repeated (beta alpha) (4) half-barrels may provide a molecular tool for dual substrate specificity. EMBO Rep.

[43] Nicholls H (2007). Sorcerer II: The search for microbial diversity roils the waters. PLoS Biol.

[44] Matney TS, Goldschmidt EP, Erwin NS, Scroggs RA (1964). A preliminary map of genomic sites for F-attachment in Escherichia coli K12. Biochem Biophys Res Commun.

[45] Norris PR, Clark DA, Owen JP, Waterhouse S (1996). Characteristics of Sulfobacillus acidophilus sp. nov. and other moderately thermophilic mineral-sulphide-oxidizing bacteria. Microbiology.

[46] Due AV, Kuper J, Geerlof A, von Kries JP, Wilmanns M (2011). Bisubstrate specificity in histidine/tryptophan biosynthesis isomerase from Mycobacterium tuberculosis by active site metamorphosis. Proc Natl Acad Sci U S A.

[47] Simon C, Daniel R (2011). Metagenomic Analyses: Past and Future Trends. Appl Environ Microb.

[48] Temperton B, Giovannoni SJ (2012). Metagenomics: microbial diversity through a scratched lens. Curr Opin Microbiol.

[49] Wellner A, Raitses Gurevich M, Tawfik DS. Mechanisms of protein sequence divergence and incompatibility. PLoS Genet. 2013;9(7):e1003665. 10.1371/journal.pgen.1003665 PMC3723536 23935519

[50] Stackebrandt E, Woese CR (1981). Towards a Phylogeny of the Actinomycetes and Related Organisms. Curr Microbiol.

[51] Ventura M, Canchaya C, Tauch A, Chandra G, Fitzgerald GF, Chater KF (2007). Genomics of Actinobacteria: tracing the evolutionary history of an ancient phylum. MMBR.

[52] Quast C, Pruesse E, Yilmaz P, Gerken J, Schweer T, Yarza P (2013). The SILVA ribosomal RNA gene database project: improved data processing and web-based tools. Nucleic Acids Res.

[53] Faulon JL, Misra M, Martin S, Sale K, Sapra R (2008). Genome scale enzyme-metabolite and drug-target interaction predictions using the signature molecular descriptor. Bioinformatics.

[54] Carbonell P, Faulon JL (2010). Molecular signatures-based prediction of enzyme promiscuity. Bioinformatics.

[55] Nagao C, Nagano N, Mizuguchi K (2014). Prediction of detailed enzyme functions and identification of specificity determining residues by random forests. PLoS One.

[56] Akanuma S, Yamagishi A (2008). Experimental evidence for the existence of a stable half-barrel subdomain in the (beta/alpha)8-barrel fold. J Mol Biol.

[57] Wierenga RK (2001). The TIM-barrel fold: a versatile framework for efficient enzymes. FEBS Lett.

