# Supplementary material for: Insights into the evolution of enzyme substrate promiscuity after the discovery of (βα)8 isomerase evolutionary intermediates from a diverse metagenome
Source: BMC Evol Biol. 2015 Jun 10;15:107. doi: 10.1186/s12862-015-0378-1 (PMC4462073; doi:10.1186/s12862-015-0378-1)
Supplement: Additional file 1: Figure S1. — Sequence alignment of functionally characterized proteins. Figure S2. E. coli trpF − and hisA − complementation assays with selected proteins. Figure S3. Catalytic activity pH dependence of HisA_Afer. Table S1. Oligonucleotides used to construct CAM1 truncated variants. Table S2. Protein sequence of synthetic genes. [file 12862_2015_378_MOESM1_ESM.doc]

**Supplementary Information for:**

**Insights into the evolution of enzyme substrate promiscuity after the discovery of (βα)_8_ isomerase evolutionary intermediates from a diverse metagenome**

Lianet Noda-García^1,4,+^, Ana L. Juárez-Vázquez^1,+^, María C. Ávila-Arcos^1,4^, Ernesto A. Verduzco-Castro^1^, Gabriela Montero-Morán^1,4^, Paul Gaytán^3^, Mauricio Carrillo-Tripp^2^ and Francisco Barona-Gómez^1,*^.

Supplementary Table 1. Forward oligonucleotide and oligonucleotide sets used to construct CAM1 truncated variants.

| **Primer ID^a^** | **Sequence 3´- 5´** |
| --- | --- |
| NdeFw | GGATTAGGAGTATACCGAGGCAGTGGAGTCTTTGG |
| **SET 1** | GTGACCGGCTTCGTCAACGGCGA tag ctc ggc gcg aag cta tac aag aag acc ccg ggc ggc gtt ATTTTCGAATTGCGC |
| i.e. Rev1.1 | GTGACCGGCTTCGTCAACGGCGA ATTTTCGAATTGCGC |
| i.e. Rev1.2 | GTGACCGGCTTCGTCAACGGCGA tag ATTTTCGAATTGCGC |
| i.e. Rev1.3 | GTGACCGGCTTCGTCAACGGCGA tag ctc ATTTTCGAATTGCGC |
| i.e. Rev1.4 | GTGACCGGCTTCGTCAACGGCGA tag ctc ggc ATTTTCGAATTGCGC |
| i.e. Rev1.5 | GTGACCGGCTTCGTCAACGGCGA tag ctc ggc gcg ATTTTCGAATTGCGC |
| i.e. Rev1.6 | GTGACCGGCTTCGTCAACGGCGA tag ctc ggc gcg aag ATTTTCGAATTGCGC |
| i.e. Rev1.7 | GTGACCGGCTTCGTCAACGGCGA tag ctc ggc gcg aag cta ATTTTCGAATTGCGC |
| i.e. Rev1.8 | GTGACCGGCTTCGTCAACGGCGA tag ctc ggc gcg aag cta tac ATTTTCGAATTGCGC |
| i.e. Rev1.9 | GTGACCGGCTTCGTCAACGGCGA tag ctc ggc gcg aag cta tac aag ATTTTCGAATTGCGC |
| i.e. Rev1.10 | GTGACCGGCTTCGTCAACGGCGA tag ctc ggc gcg aag cta tac aag aag ATTTTCGAATTGCGC |
| i.e. Rev1.11 | GTGACCGGCTTCGTCAACGGCGA tag ctc ggc gcg aag cta tac aag aag acc ATTTTCGAATTGCGC |
| i.e. Rev1.12 | GTGACCGGCTTCGTCAACGGCGA tag ctc ggc gcg aag cta tac aag aag acc ccg ATTTTCGAATTGCGC |
| i.e. Rev1.13 | GTGACCGGCTTCGTCAACGGCGA tag ctc ggc gcg aag cta tac aag aag acc ccg ggc ATTTTCGAATTGCGC |
| i.e. Rev1.14 | GTGACCGGCTTCGTCAACGGCGA tag ctc ggc gcg aag cta tac aag aag acc ccg ggc ggc ATTTTCGAATTGCGC |
| i.e. Rev1.15 | GTGACCGGCTTCGTCAACGGCGA tag ctc ggc gcg aag cta tac aag aag acc ccg ggc ggc gtt ATTTTCGAATTGCGC |
| **SET 2** | CTTCCGCGGTAATAACCTGCA cgg gac atg ctc ttg cgg aag tgt gac cgg ctt cgt cac cgg ATTTTCGAATTGCGC |
| **SET 3** | GACCTTCTAGACGTATTTGAC cgc cta gac cgt tgt gta ccc taa ctt ccg cgg tag tag cct ATTTTCGAATTGCGC |
| **SET 4** | CACTAGTATGGCTCGCGGGACAC tag cgc agc ccc cca tag cgt gcg gac ctt ctg gac gtg ttt ATTTTCGAATTGCGC |
| **SET 5** | CTACCCTACTACGTACCAGGCTTA gac gtg gac gac ttt ttt cac acg ctg gtg tgg ctc gcg gga ATTTTCGAATTGCGC |

^a^ Primers named Rev1.1 – Rev1.15 are an example of those oligonucleotide contained in SET1. The five oligonucleotide sets were assembled by the resin splitting method briefly described in Material and Methods.

Supplementary Table 2. Protein sequence of synthetic genes

| **Protein Identifier** | **Sequence** |
| --- | --- |
| C7LZ82 | MELVVAVDLLDGEAVRLRQGRFDAPRRFGDPRRYVEGALDAGARWFHVVDLDRARTPGDRRNADMVGDLIALVREAGGSVEVGGGVRTARDIAELLETGASRVVLGTALVEETLDELDPEHLVAALDYRRGDEGALEVVVSAWERSGGRRLLEASARLFARGVRTQLLTDVGRDGMASGPDLGTYQGLCEVVPVEVIASGGVASADDLAKLAAIRGARGAVWGAVVGTALLDGSLGFDEALSACRA |
| CAM1 | MFALLPAVDVAEGRAVRLVQGAAGTETDYGDPVAAARSWVEQGAPWIHLVDLDAAFGRGDNRAVVHRVVQAVGGDVHVEVAGGIRDDASLDAALASGASRAVIGTAAMENPDWVRSAIDRYGDRLAVSLDVRGHTLAARGWTTEGGDLFETIGRLDADGCARYIVTDVEKDGMMHGPNLHLLKKVCDHTERPVIASGGIARLEDLHKLADLATHGIEGAIIGRALYENAFTLAEAVAAIE |
| CAM2 | MFALLPAVDVAEGRAVRLVQGAAGTETDYGDPVAAARSWVEQGAPWIHLVDLDAAFGRGDNRAVVHRVVQAVGGDVHVEVAGGIRDDASLDAALASGASRAVIGTAAMENPDWVRSAIDRYGDRLAVSLDVRGHTLAARGWTTEGGDLFETIGRLDADGCARYIVTDVEKDGMMHGPNLHLLKMCAITRNGRSSRKL |

Supplementary Figure 1. Sequence alignment of functionally characterized proteins. A) Alignment of HisA, CAM1 and PriA; N-PBS motif is marked with a black rectangle. B) C-terminal regions of CAM2 characterized variants.

Supplementary Figure 2. *E. coli trpF^-^ and hisA^-^*complementation assays with selected proteins. CAM2 low *in vivo* activity is highlighted with a white circle. CAM_215a and b posses the same sequence.


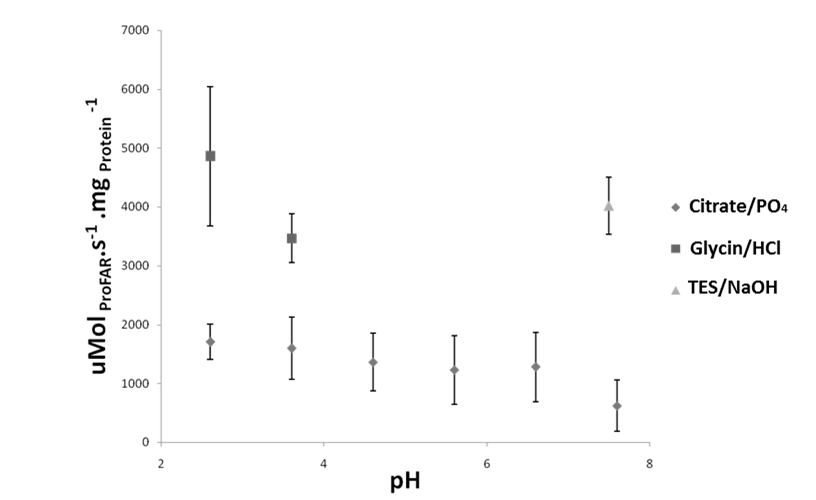


Supplementary Figure 3. Catalytic activity pH dependence. In order to determine the ProFAR activities of HisA_Afer (C7LZ82) the enzymatic assay described in Methods was modified. ProFAR isomerase activity was determined at a range of pH values (2.6 to 7.6) using the buffer systems Glycin/HCl, Citrate/PO4 and TES/NaOH at 50mM. Lower activity in Citrate/PO_4_ system is due to competition of citrate and phosphate ions with the phosphorylated substrate ProFAR.
